# A Transcriptome Analysis of *Poncirus trifoliata*, an Aurantioideae Species Tolerant to Asian Citrus Psyllid, Has Identified Potential Genes and Events Associated with Psyllid Resistance

**DOI:** 10.3390/insects15080589

**Published:** 2024-08-02

**Authors:** Xinyou Wang, Haoran Ji, Leijian Zhong, Wei Zeng, Zhigang Ouyang, Ruimin Li

**Affiliations:** 1 College of Life Sciences, Gannan Normal University, Ganzhou 341000, China; xinyouwang@gnnu.edu.cn (X.W.); jihaoran58570@outlook.com (H.J.);; 2 National Navel Orange Engineering Research Center, Ganzhou 341000, China; 3 Jiangxi Provincial Key Laboratory of Pest and Disease Control of Featured Horticultural Plants, Ganzhou 341000, China

**Keywords:** *Poncirus trifoliata*, *Diaphorina citri*, transcriptome, insect resistance, transcriptional

## Abstract

**Simple Summary:**

Our study investigated the molecular mechanism of co-cultivated *Citrus sinensis* in diminishing the resistance of *Poncirus trifoliata* to the Asian Citrus Psyllid (ACP). The presence of *C. sinensis* was found to weaken the resistance of *P. trifoliata* to ACP, shown by a significant increase in ACP survival after 14 days of incubation. RNA-seq analysis revealed differentially expressed genes (DEGs) between control samples and those under co-cultivation with *C. sinensis*, with significant changes in metabolic processes. Further analysis indicated that *P. trifoliata* co-cultivation with *C. sinensis* significantly impacted the expression of many genes, including those involved in polysaccharide metabolic processes, metabolic pathways, biosynthesis of secondary metabolites, and phenylpropanoid biosynthesis. The study also identified a hub gene, *Ptrif_0004s_1029*, a *NAC* gene from transcription factor genes, appearing crucial in transcriptional regulation related to potential ACP resistance. Additionally, DEGs related to ACP feeding were also identified. Our research has elucidated the molecular mechanisms that underlie the resistance of *P. trifoliata* to the ACP and identified potential genes associated with this resistance.

**Abstract:**

Citrus huanglongbing (HLB) is a devastating disease for citrus production, largely caused by the Asian citrus psyllid (ACP). *Poncirus trifoliata* exhibits high resistance to ACP; however, this resistance is weakened when *C. sinensis* is co-cultivated. This study aimed to identify the differentially expressed genes (DEGs) during ACP feeding and to uncover potential ACP resistance genes in *P. trifoliata*. In comparison to independent cultivation, 1247 and 205 DEGs were identified in *P. trifoliata* when co-cultivated with *C. sinensis* after 7 and 14 days, respectively. Analysis of enriched Gene Ontology categories revealed that DEGs were significantly associated with the cell wall, glucometabolic activities, and secondary metabolites. Additionally, these genes were found to be involved in phytohormone signaling, cell wall metabolism, redox state homeostasis, and secondary metabolites, as well as a number of transcription factor genes (TFs). Furthermore, we examined the impact of the ACP feeding factor on the gene expression patterns in *P. trifoliata*. Results showed an increase in the JA signaling pathway and various TFs. The RNA-seq results were verified using reverse transcription quantitative PCR. Our findings shed light on the molecular basis of ACP resistance in *P. trifoliata* and identified potential genes associated with this resistance.

## 1. Introduction

Cultivation of citrus is widespread in more than a hundred countries and regions, making it a significant global fruit crop [1,2]. The citrus industry is also subject to various environmental pressures, including infection by pathogens, as well as cold and drought stress [2,3]. Citrus huanglongbing (HLB) is a severe affliction in citrus production, the cause of which is the bacterium *Candidatus* Liberibacter asiaticus (*C*Las) [4]. This disease is mainly spread in the field by the Asian citrus psyllid (*Diaphorina citri*, ACP) [5]. *Poncirus trifoliata* is an optimal rootstock for cultivated citrus varieties like *C. sinensis* and *C. reticulata* [6]. *P. trifoliata* is renowned for its remarkable tolerance to cold and dry stresses [7]. In addition to demonstrated immunity to citrus tristeza virus (CTV) [8], citrus nematodes [9], and *Phytophthora* pathogens [10,11], *P. trifoliata* is also noted for its tolerance to HLB and resistance to ACP [12].

Plant cells possess the ability to detect insects feeding through the use of damage-associated molecular patterns (DAMPs), similar to how pathogen-associated molecular patterns (PAMPs) are recognized [13]. Phytohormone regulatory mechanisms incorporate multiple insect recognition signals. Plant–insect interactions are mediated by the jasmonic acid (JA) signaling pathway, which is triggered when insects feed and leads to heightened levels of JA-Ile and the degradation of the JAZ proteins [14]. Subsequently, JA responsive genes are activated in order to defend against the insect [15]. For example, cotton plants displayed an elevation in JA levels after *Spodoptera exigua* feeding, which subsequently resulted in the synthesis of secondary metabolites to impede the growth of *S. exigua* [16]. It has been observed that exogenous abscisic acid (ABA) can significantly enhance the resistance of rice to brown planthopper [17]. Additionally, ABA functions as the activator of the JA signaling pathway in *Arabidopsis*–*Pieris rapae* interactions [18]. Moreover, insect infestation has an impact on the levels of auxin, gibberellins, cytokinin, salicylic acid (SA), and brassinosteroid, and the JA-signaling pathway forms the center of the cross-talk between the various phytohormone signaling pathways [19]. However, resistance to phloem feeders was demonstrated to be mediated by salicylic acid (SA) via a signaling pathway that is independent of JA [20,21]. Apart from phytohormone signaling pathways, numerous insect resistance genes have been identified in the genome of plants. For instance, *H* and *Dn* genes in *Triticum aestivum* [22,23,24,25], *Bph* and *Gm* genes in *Oryza sativa* [26,27,28,29], *Mi-1,2* in *Solanum lycopersicum* [30], Rag genes in Glycine max [31,32,33], *Vat* gene in *Cucumis melo* [34], and *AIN* gene in *Medicago truncatula* [35] were continually identified.

Evidence from prior studies has indicated that *C. macrophylla* can weaken the resistance of *P. trifoliata* to ACP to some degree [12]. Our study revealed that the presence of *C. sinensis* substantially diminished the resistance of *P. trifoliata* to ACP, leading to a significant rise in the survival rate of ACP. To elucidate the mechanism and pinpoint ACP-resistant genes in *P. trifoliata*, we conducted an RNA-seq analysis to detect differentially expressed genes (DEGs) and revealed their functional roles. Our findings enlighten the molecular basis of the ACP resistance in *P. trifoliata* and identify potential genes that can be used in breeding citrus for ACP resistance.

## 2. Materials and Methods

### 2.1. Plant and Insect Materials

Two-year-old *P. trifoliata* accession “YA-1” and *C. sinensis* “Newhall” plants were cultivated in separate greenhouses in controlled conditions (28 ± 3 °C, relative humidity of 50 ± 10%, and a regular photoperiod of 14 h light/10 h dark). Additionally, ACP was collected from a citrus orchard in Xinfeng County, Ganzhou City, Jiangxi Province, China and reared on *Murraya paniculata* in the insectarium of Gannan Normal University.

### 2.2. Experiment Design

In this study, 6 two-year-old *P. trifoliata* accession “YA-1” plants were arranged in pairs with 6 *C. sinensis* “Newhall” in a greenhouse (the Stress Treatment Group, or S Group), and another 6 two-year-old *P. trifoliata* accession “YA-1” plants were placed in separate greenhouses (the control group, or CK Group). The number of plants in each S group is consistent, with 6 *P. trifoliata* and 6 *C. sinensis* plants. In addition, 6 *P. trifoliata* plants were used for each CK group. After 7 days, 20 adult ACPs were introduced to the fresh branches (10–15 cm top of the plants) of each *P. trifoliata* plant in individual nylon cages for 14 days. The survival numbers of adult ACPs were counted at 7 days and 14 days (6 biological replications). In our study, *P. trifoliata* plants that were used for enumerating the survival numbers of ACPs would not be used for RNA-seq samples collection. Leaf samples for RNA-seq were taken from another parallel group of *P. trifoliata* plants.

### 2.3. RNA-Seq Analyses of Stress Treatment and Control Group

Fresh leaves from three randomly selected *P. trifoliata* plants were collected and immediately frozen with liquid nitrogen for both the stress treatment and control groups at 0, 7, and 14 days. The same sample was used for day 0 of stress treatment and control groups. Total RNA was extracted using the Plant RNA Kit (Omegabiotek, Guangzhou, China) and the concentration and quality of the RNA was assessed using a NanoDrop 2000 (Thermo Fisher Scientific, Wilmington, DE, USA). Qualified samples were then sent to BioMarker (BioMarker, Beijing, China) for cDNA library construction and RNA-seq analysis. Libraries were prepared using the NEBNext UltraTM RNA Library Prep Kit for Illumina (NEB, Ipswich, MA, USA) and sequenced using the Illumina HiSeq X Ten platform to generate 150 bp paired-end reads. The raw reads were filtered by a trimmomatic and then aligned to the *P. trifoliata* reference genome [6] using HISAT2 version 2.2.1 [36]. Read counts of genes were quantified using StringTie version 2.1.5 [37]. Differently expressed genes (DEGs) analysis was generated by DESeq2 packages in Omicshare platform (www.omicshare.com, accessed on 22 August 2023) with adjusted *p* value less than 0.05. Venn analysis for different gene clusters was performed using EVenn v1.0 and Venny v2.1 [38,39]. A heatmap was drawn using the TBtools toolkit version 1.098 [40].

### 2.4. Gene Ontology (GO), KEGG Enrichment Analysis and MapMan Visualization

DEGs were applied to functional analysis, and GO and KEGG enrichment analyses were conducted using the “gogseasenior” and “pathwaygseasenior” tools on Omicshare platform (www.omicshare.com, accessed on 22 August 2023). The pathways and biological processes in which DEGs were involved were then visualized via MapMan 3.5.1R2 [41].

### 2.5. Co-Expression Network Construction

The Weighted Gene Co-expression Network Analysis (WGCNA) package [42] in R was used to construct a co-expression network. In brief, the count values of all genes were imported into WGCNA and genes with loss of expression or missing values were filtered. Pearson correlation was used to calculate the similarity between genes, and a soft threshold was determined to ensure that the linkages between genes obeyed scale-free networks. Hierarchical clustering was used to generate various gene modules, and key modules were filtered based on their correlation with sample characteristics. Finally, genes with a high connectivity degree were identified as hub genes.

### 2.6. Quantitative Real-Time PCR (qRT-PCR)

To validate the RNA-seq data, qRT-PCR was employed. Total RNA was extracted and reverse transcribed using TransScript^®^ One-Step gDNA Removal and cDNA Synthesis SuperMix kit (Transgen, Beijing, China). Subsequently, PCR was performed on an ABI StepOne fluorescence ration PCR instrument with TransStart^®^ Green qPCR SuperMix (Transgen, Beijing, China). The expression of the candidate genes was then calculated using the 2^−ΔΔCT^ method [43]. All primers used in this study were listed in Table S6.

### 2.7. Statistical Analysis

The differences in mean survival numbers were analyzed using a two-tailed Student’s *t*-test in SPSS 25.0 between the S and CK Groups.

## 3. Results

### 3.1. C. sinensis Attenuates the Resistance of P. trifoliata to ACP

**Figure 1:**
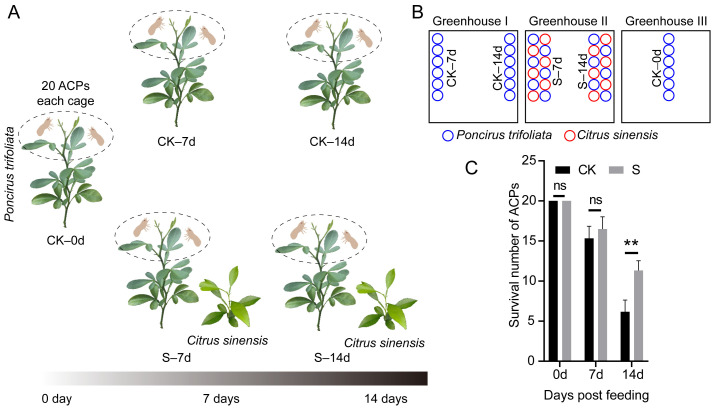
Experimental design and survival statistics of Asian citrus psyllid (ACP). (**A**) Design of experimental group and control group. (**B**) Plants distribution in the greenhouse. (**C**) Survival statistics of ACPs. The experimental group, also referred to as the stress group, is denoted by “S” while the control group is denoted by “CK”. The sample names are labeled under the schematic diagram, which are CK–0d, CK–7d, CK–14d, S–7d, and S–14d, respectively. The symbol “ns” denotes nonsignificant differences between samples at a significance level of 0.05, whereas “**” signifies significant differences between samples at a significance level of 0.01.

To ascertain whether *C. sinensis* reduces the immunity of *P. trifoliata* to ACP, we cultivated *C. sinensis* plants in conjunction with *P. trifoliata* in a separate plant growth chamber, while the control group was solely composed of *P. trifoliata*, grown in a different independent plant growth chamber (Figure 1A). Six biological replicates of *P. trifoliata* plants were incubated for a period of two weeks, each containing twenty ACPs. The survival number of ACPs showed no significant when incubated for 7 days, but when incubated for 14 days, the results were incredibly significant between S samples and CK samples (Figure 1B). The CK samples revealed a decrease in the number of ACP survivors, suggesting that *C. sinensis* could weaken the resistance of *P. trifoliata* to ACP (Figure 1B).

### 3.2. RNA-Seq Analysis

**Figure 2:**
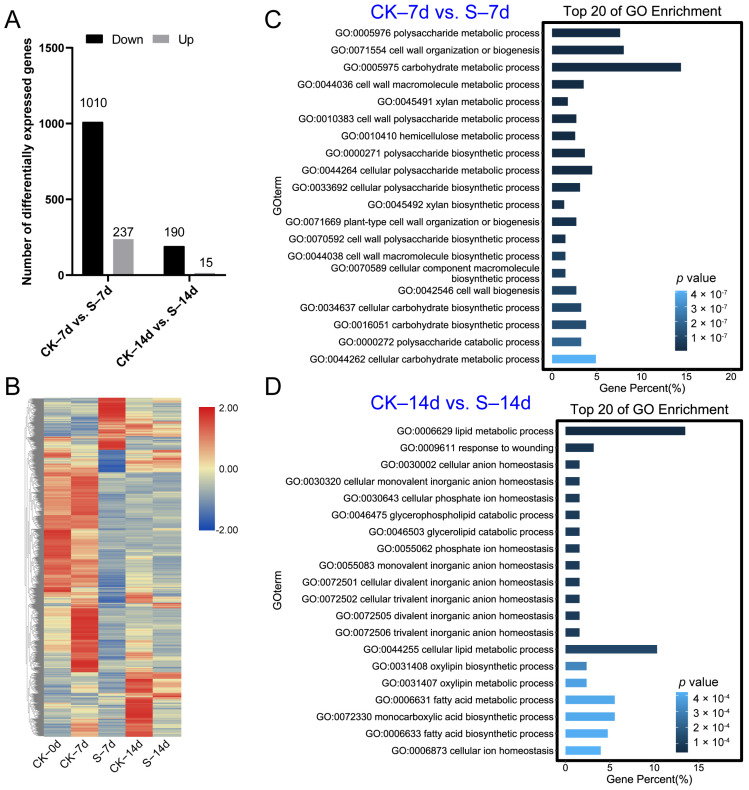
RNA-seq analysis and gene ontology enrichment of differential expressed genes (DEGs) between S and CK samples. (**A**) Number of DEGs. (**B**) Heatmap of DEGs. (**C**) GO enrichment analysis of DEGs at 7 days. (**D**) GO enrichment analysis of DEGs at 14 days. “CK–7d vs. S–7d” and “CK–14d vs. S–14d” refer to the comparison of the CK samples with the S samples 7 days and 14 days after ACPs feeding, respectively.

To uncover the transcriptional regulation between the S samples and the CK samples, samples at each time point were obtained for RNA-seq analysis. Our study revealed that compared to the CK samples, the S samples had 1010 down-regulated and 237 up-regulated genes at 7 days, and 190 down-regulated and 15 up-regulated genes at 14 days (Figure 2A and Table S1). The expression pattern of all DEGs suggested that stress significantly impacted the expression of many genes (Figure 2B).

Analysis of DEGs between the CK and S samples at 7 and 14 days revealed significant enrichment of metabolic processes such as “polysaccharide metabolic process”, “cell wall organization or biogenesis”, “carbohydrate metabolic process”, “cell wall macromolecule metabolic process”, and “xylan metabolic process” (Figure 2C and Table S3). Furthermore, at 14 days, GO enrichment analysis of DEGs indicated notable influence on processes such as “lipid metabolic process”, “response to wounding”, “cellular anion homeostasis”, “cellular monovalent inorganic anion homeostasis”, and “cellular phosphate ion homeostasis” (Figure 2D and Table S2).

### 3.3. Functional Analysis of DEGs between the CK and S Samples

**Figure 3:**
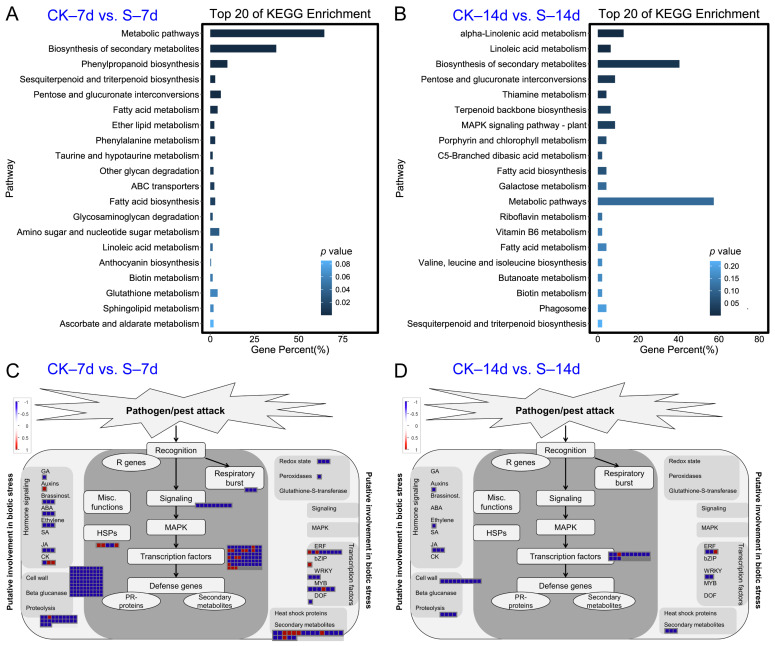
KEGG enrichment analysis and functional classification of DEGs between S and CK samples post ACP feeding. (**A**) KEGG enrichment analysis of DEGs at 7 days. (**B**) KEGG enrichment analysis of DEGs at 14 days. (**C**) Functional classification of DEGs involved in biotic stresses at 7 days post ACP feeding. (**D**) Functional classification of DEGs involved in biotic stresses at 14 days post ACP feeding.

In addition, KEGG pathway enrichment analysis of DEGs revealed the presence of several pathways such as “Metabolic pathways”, “Biosynthesis of secondary metabolites”, “Phenylpropanoid biosynthesis”, “Sesquiterpenoid and triterpenoid biosynthesis”, “Pentose and glucuronate interconversions”, “Alpha-Linolenic acid metabolism”, “Linoleic acid metabolism”, and “Thiamine metabolism” (Figure 3A,B and Table S3). MapMan visualization of the DEGs involved in biotic stress revealed that genes related to “Hormone signaling”, “Redox state”, “Signaling”, “Secondary metabolites”, “Cell wall”, “Respiratory burst” and other categories were significantly decreased in S samples at 7 days, whereas, much fewer DEGs were observed in S samples at 14 days (Figure 3C,D and Table S4). Analysis of phytohormone signaling genes revealed that, at 7 days, there were various DEGs: one gibberellin-, one cytokinin-, three ABA-, three Ethylene- and three JA-related genes (including two 13-lipoxygenase genes, *LOX*, and one allene oxidase cyclase gene) were down-regulated, while one Auxin- and two cytokinin-related genes were up-regulated (Figure 3C). At 14 days, one Auxin-, one Ethylene- and three JA-related DEGs associated with phytohormone signaling were down-regulated (Figure 3D).

**Figure 4:**
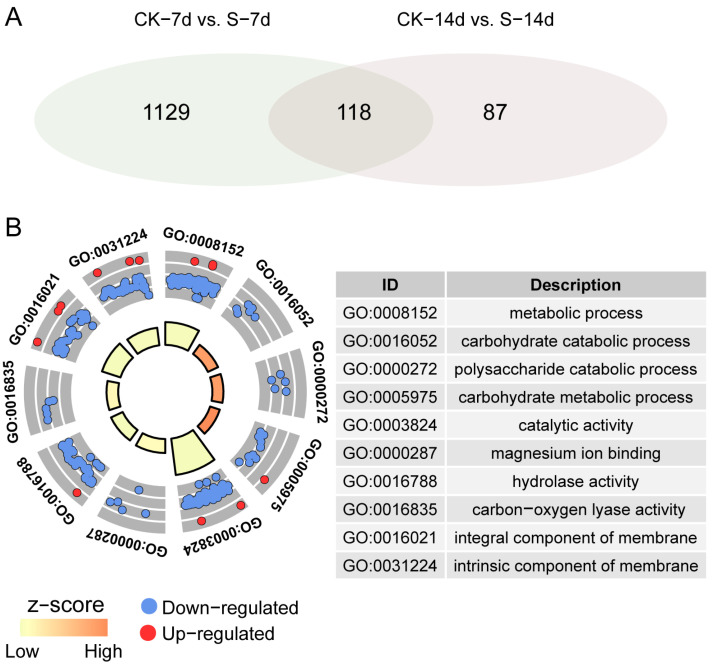
Identification and GO enrichment analysis of common DEGs between S and CK samples at 7 days and 14 days post ACP feeding. (**A**) Venn diagram analysis of common DEGs. (**B**) GO enrichment analysis of common DEGs. Blue dots represent down-regulated genes, while red dots represent up-regulated genes.

We identified 118 DEGs, which were common between 7 and 14 days (Figure 4A), and 10 GO terms were enriched, particularly the “carbohydrate catabolic process” and “catalytic activity” terms, which were significantly affected (Figure 4B).

**Figure 5:**
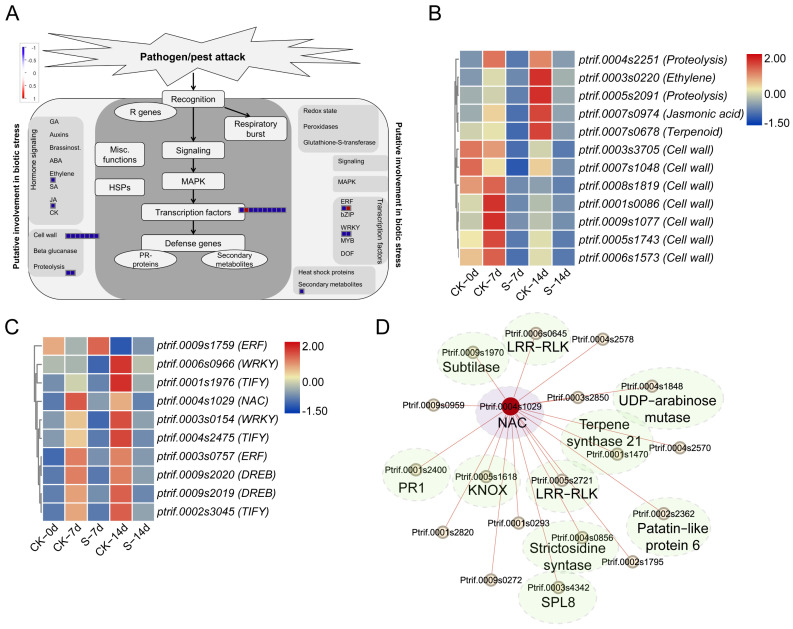
Functional analysis of common DEGs between S and CK samples at 7 days and 14 days post ACP feeding. (**A**) Functional classification of common DEGs. (**B**) Biotic stress related DEGs. (**C**) Differentially expressed transcription factor genes. (**D**) Hub gene generated using WGCNA. *NAC* signifies NAM, ATAF1/2, and CUC2 transcription factor, PR1 denotes Pathogenesis related protein-1, KNOX represents Knotted1-like homeobox transcription factor, LRR-RLK indicates Leucine-rich repeat receptor-like protein kinase, and SPL8 stands for SQUAMOSA-promoter binding protein-like transcription factor.

Functional analysis of the DEGs indicated various genes involved in biotic stress had been disrupted (Figure 5). Notably, genes associated with phytohormone, cell wall metabolism, proteolysis, secondary metabolites, and ten transcription factor genes (including *DREB*, *ERF*, *NAC*, *TIFY*, and *WRKY*) were observed (Figure 5B,C). Additionally, we conducted an investigation into the hub gene involved in transcriptional regulation. Co-expression analysis revealed several modules related to ACP resistance (Figure S1), with one such network depicted in Figure 5D. The hub gene was identified as *Ptrif_0004s_1029*, a *NAC* gene belonging to the aforementioned transcription factor genes. Genes associated with *Ptrif.0004s.1029*, such as *PR-1*, *LRR-RLK*, *Patatin-like protein 6*, *terpene synthase 21*, *subtilase*, *strictosidine synthase*, and *SPL8*, were involved in biotic stress and development (Figure 5D).

### 3.4. Functional Analysis of DEGs Related with ACP Feeding

**Figure 6:**
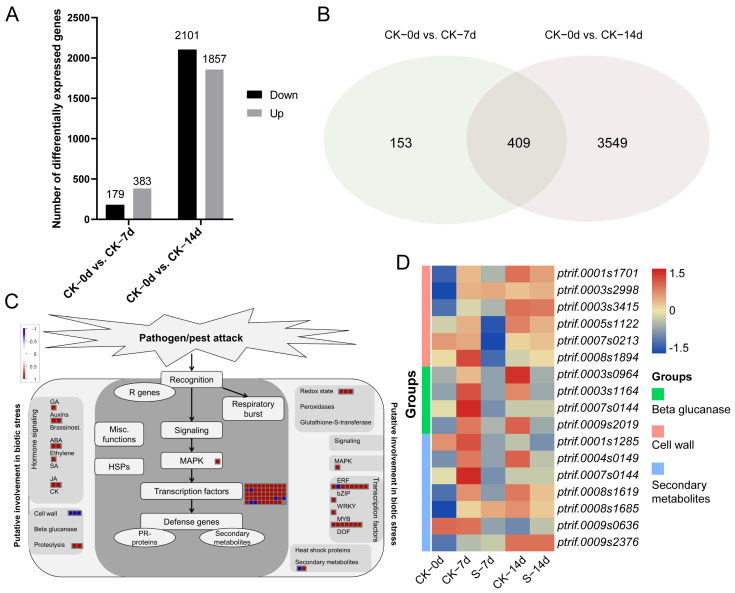
Functional analysis of DEGs between CK samples at different points in time. (**A**) Number of DEGs. (**B**) Venn diagram analysis of common DEGs. (**C**) Functional classification of common DEGs. (**D**) Heatmap of Biotic stress related DEGs. “CK–0d vs. CK–7d” and “CK–0d vs. CK–14d” refer to the comparison of the CK samples 7 days and 14 days after ACPs feeding with control samples, respectively.

To identify genes associated with ACP feeding, DEGs between CK samples at various time points was determined. A total of 562 DEGs were identified when comparing CK–7d to CK–0d samples, with 179 down-regulated and 383 up-regulated (Figure 6A). Additionally, 3958 DEGs were identified when comparing CK–14d to CK–0d samples, comprising of 2101 down-regulated and 1857 up-regulated (Figure 6A). The two comparisons revealed 409 DEGs in common (Figure 6B), and these DEGs were associated with cell wall, beta glucanase, redox state, and secondary metabolites processes included eight phytohormone related genes, three cell wall metabolism genes, and forty-nine transcription factor genes (Figure 6C). Notably, the expression of these candidate genes showed a marked up-regulation in CK–7d samples and down-regulation in S–7d samples (Figure 6D).

GO enrichment analysis revealed that processes such as “aminoglycan catabolic process”, “chitin metabolic process”, “amino sugar catabolic process” and “response to stress” were significantly more abundant in DEGs between CK–7d and CK–0d samples (Figure S2A), while “protein phosphorylation”, “photosynthesis, light harvesting”, “oxidation-reduction process”, “lignin biosynthetic process” were enriched in DEGs between CK–14d and CK–0d samples (Figure S2B). In addition, KEGG enrichment analysis demonstrated that pathways such as “Biosynthesis of secondary metabolites”, “Phenylpropanoid biosynthesis”, “Fatty acid metabolism”, “Peroxisome” and “Plant hormone signal transduction” were enriched in DEGs between CK–7d and CK–0d samples (Figure S3A). Furthermore, “Biosynthesis of secondary metabolites”, “Photosynthesis”, “Starch and sucrose metabolism”, and “Plant hormone signal transduction” were also enriched in DEGs between CK–14d and CK–0d samples (Figure S3B).

### 3.5. Validation RNA-Seq Using qRT-PCR

**Figure 7:**
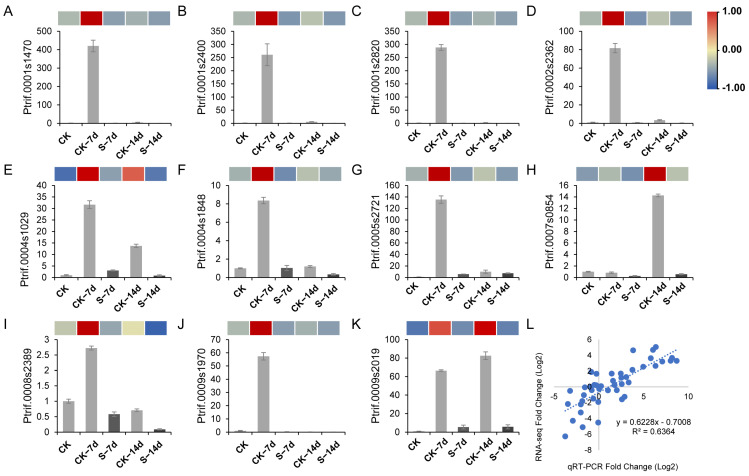
Correlation analysis of RNA-seq and qRT-PCR results of candidate genes. Expression profiles of (**A**) *Ptrif.0001s1470*, (**B**) *Ptrif.0001s2400*, (**C**) *Ptrif.0001s2820*, (**D**) *Ptrif.0002s2362*, (**E**) *Ptrif.0004s1029*, (**F**) *Ptrif.0004s1848*, (**G**) *Ptrif.0005s2721*, (**H**) *Ptrif.0007s0854*, (**I**) *Ptrif.0008s2389*, (**J**) *Ptrif.0009s1970*, and (**K**) *Ptrif.0009s2019*. (**L**) Pearson correlation analysis.

To validate the reliability of RNA-seq in this study, qRT-PCR was employed and eleven candidate genes were randomly selected for this purpose. The qRT-PCR results were highly correlated with the RNA-seq results, as demonstrated by *Ptrif.0009s2019*, which was up-regulated in both CK–7d and CK–14d samples as observed in both qRT-PCR and RNA-seq data (Figure 7 and Table S5).

## 4. Discussion

*P. trifoliata* exhibits high resistance to ACP [6]. In this study, RNA-seq was employed to detect potential ACP resistance genes in *P. trifoliata*, which included phytohormone related genes, cell wall metabolic genes, fatty acid biosynthesis genes, secondary metabolism genes, and redox homeostasis related genes.

Plant–plant interactions are pivotal in elucidating community structure and ecosystem functioning, with the exchange of chemical signals, particularly volatile organic compounds (VOCs), emerging as a key interaction mechanism [44,45]. VOCs play a crucial role in mediating plant–plant and plant–pest interactions, especially in plant defense against pests [46,47]. When plants are attacked by pests, they may release VOCs as a distress signal, attracting predatory insects that indirectly contribute to pest defense [48]. Moreover, VOCs can directly repel pests or interfere with their physiological functions [49]. Additionally, VOCs emitted by damaged plants can trigger defensive reactions in neighboring plants, enhancing their resistance against pests [50]. However, planting pest-susceptible species alongside pest-resistant ones might compromise the pest resistance of the latter, possibly due to the disruptive effects of VOCs from the susceptible plants on the metabolic processes of the resistant plants [12]. For instance, the presence of *C. macrophylla* can heighten the susceptibility of *P. trifoliata* to ACPs [12]. In this study, we observed that the coexistence of *C. sinensis* could diminish the resistance of *P. trifoliata* to ACPs, likely through intricate interactions of plant VOCs, underscoring the potential benefits of this mechanism in breeding citrus varieties with enhanced ACP resistance.

Moreover, phytohormone signaling pathways are essential for plant–insect interactions, with JA signaling pathway being the main mediator of plant system resistance to insects [51]. The 13-lipoxygenase catalyzed transformation of α-Linolenic acid to 13-Hydroperoxolenic acid is a significant step in the endogenous synthesis of JA [52]. Enhanced expression of *GhLOX2*, a 13-lipoxygenase gene, increased the tolerance of cotton to *Verticillium dahlia* [53]. *TomloxD*, a 13-Lipoxygenase Gene from tomato, when overexpressed, provides resistance to *Cladosporium fulvum* [54]. The 13-lipoxygenase mediated JA pathway to bolster resistance to chewing and piercing-sucking herbivores in rice [55]. Our research revealed that the 13-lipoxygenase genes were heightened in response to ACP feeding, yet weakened in the presence of *C. sinensis*, potentially reducing the immunity of *P. trifoliata* to ACP. Additionally, the allene oxide cyclase is a significant factor in JA signaling and suppresses of plant growth caused by herbivory [56]. In this study, the expression of the allene oxidase cyclase gene, which is involved in the biosynthetic of JA, was reduced in the presence of *C. sinensis*. The results showed that ACP, being a phloem feeder, had a significant impact on JA signaling, while no SA signaling related genes were affected. The 1-aminocyclopropane-1-carboxylic acid (ACC) synthase (ACS) and ACC oxidase (ACO) are key enzymes in ethylene biosynthesis [57]. It has been established in previous research that ethylene signaling is involved in the plant’s response to herbivory and this signaling requires the induction of JA in conjunction [58,59,60]. We observed an intriguing result in which the *ACS* and *ACO* genes in *P. trifoliata* was induced in response to ACP feeding, yet weakened upon exposure to *C. sinensis*, potentially playing a role in enhancing the resistance of *P. trifoliata* to ACP.

Activated TRICHOME BIREFRINGENCE-LIKE37 is capable of acetylating cell walls, thereby increasing resistance to herbivores [61]. Insect feeding causes a modulation in the expression of genes encoding cellulose and pectin enzymes that are associated with the cell wall [62,63]. It is noteworthy that many genes associated with the cell wall were down-regulated when *P. trifoliata* was co-cultivated with *C. sinensis*, however, these genes were not induced upon ACP feeding alone in our study. A possible explanation is that ACP belongs to piercing-sucking insects, so genes related to the cell wall, such as cellulose and pectin, would not be induced. On the other hand, chewing insects often cause a wound, thus inducing cell wall related genes [64]. The maintenance of redox homeostasis is critical for sustaining normal cellular metabolism in plants. In cells, Superoxide dismutase (SOD) eliminates negative ions of oxygen and creates hydrogen peroxide, which is then broken down by enzymes such as catalase, ascorbate peroxidase, glutathione peroxidase, thioredoxin, and others [65]. Our research revealed an upsurge in the expression of multiple genes, such as superoxide dismutase, H-type thioredoxin, GDP-D-mannose-epimerase, GDP-L-galactose, and MPBQ-methyltransferase, during ACP feeding. The altered gene expression patterns suggested that ACP consumption disturbed the equilibrium of *P. trifoliata*.

The soybean aphid has the potential to disrupt the biosynthesis of fatty acids, thus causing a reduction in the production of polyunsaturated fatty acids in soybean. During the soybean aphid infection, the activity of fatty acid desaturase 2 (FAD2) and FAD6 were found to be reduced [66]. During ACP feeding, two *FAD2* genes in *P. trifoliata* were observed to be suppressed in this study. Moreover, in our research, we observed that two *long-chain acyl-CoA synthetase* (*ACSL*) genes were activated when ACP was provided, yet their expression was reduced when C. sinensis was present. Additionally, we found that DEGs associated with flavonoid biosynthesis were increased upon ACP feeding such as genes encoding phenylalanine ammonia lyase, 4-coumarate: CoA ligase, chalcone synthase, chalcone isomerase. Flavonols may be able to shield plants from plant-feeding insects by impacting their feeding behaviors [67]. After being fed on by stink bugs, soybean responded by significantly increasing its flavonoid levels as a form of protection [68]. Consequently, ACP feeding could stimulate the expression of genes related to fatty acid biosynthesis and flavonoid biosynthesis, which could act as resistance genes against ACP.

Further, the ACP feeding was observed to influence the expression of numerous transcription factor genes such as *ERF*, *WRKY*, *NAC*, *TIFY*, and *DREB*. Except for one ERF TF, *ptrif.009s1759*, which displayed a decrease in expression, the other TFs were up-regulated. A hub *NAC* gene, *ptrif.0004s1029*, was recognized through co-expression analysis and was found to be responsible for controlling the expression of a cluster of genes, comprising *PR-1*, *SPL8*, *LRR-RLK*, and *KNOX*. *NAC* TFs are a particular type of protein found in plants that play a role in growth, development, and the response to both abiotic and biotic stresses [69]. *NAC* proteins also play an essential part in plants response to insects feeding [70]. The TFs generated in this study may be significant potential contributors to resistance to ACP in *P. trifoliata*.

## 5. Conclusions

**Figure 8:**
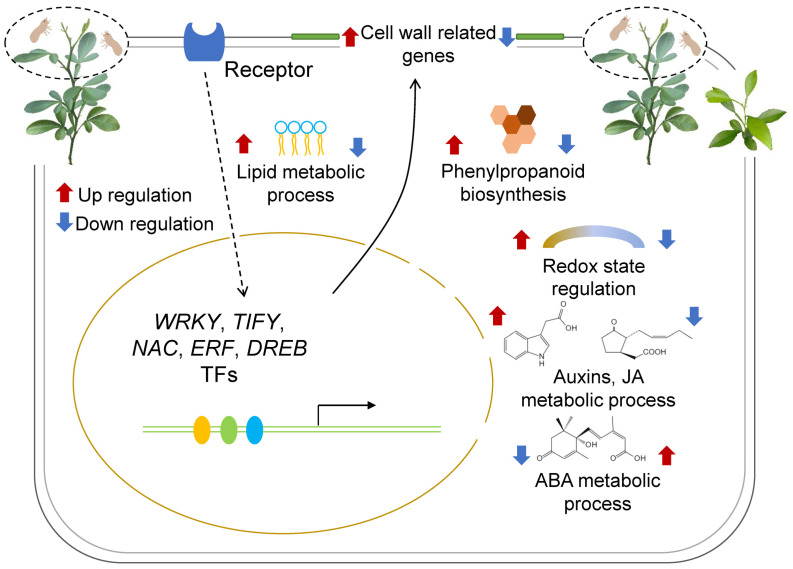
A possible functional model of *C. sinensis* attenuates the resistance of *P. trifoliata* to ACP feeding. Up-regulation biological processes are indicated by red arrows, while blue arrows signify down-regulation biological processes.

In summary, we identified a plethora of DEGs and associated biological processes and pathways involved in the resistance of *P. trifoliata* to ACP. Cell wall-related processes, fatty acid biosynthesis, phenylpropanoid biosynthesis, redox homeostasis, JA and ethylene signaling pathways, and various TF genes including *NAC*, *ERF*, *WRKY*, *TIFY*, and *DREB* were up-regulated (Figure 8) in the presence of ACP but weakened in the presence of *C. sinensis*, thereby reinforcing the notion that these genes are involved in the defense of plants against ACP. Our research revealed novel insights into the ACP resistance of *P. trifoliata*, which should be beneficial for ACP resistance citrus breeding.

## Data Availability

All data generated and analyzed during this study are included in this published article. The original RNA-seq data have been uploaded to the NCBI_SRA database (PRJNA806490).

## Supplementary Materials

The following supporting information can be downloaded at: https://www.mdpi.com/article/10.3390/insects15080589/s1, Figure S1: Co-expression network construction using WGCNA analysis. (A) Gene clustering. (B) Module-trait relationships; Figure S2: Gene ontology enrichment of differential expressed genes (DEGs) between CK samples at different points in time. (A) DEGs between CK–7d and CK–0d samples. (B) DEGs between CK–14d and CK–0d samples; Figure S3: KEGG enrichment of differential expressed genes (DEGs) between CK samples at different points in time. (A) DEGs between CK–7d and CK–0d samples. (B) DEGs between CK–14d and CK–0d samples; Table S1. Differential expressed genes identified in this study; Table S2. GO enrichment analysis of differential expressed genes in this study; Table S3. KEGG enrichment analysis of differential expressed genes in this study; Table S4. MapMan analysis of differential expressed genes in this study; Table S5. Relative expression and FPKM value of candidate genes by qRT-PCR; Table S6. Primers for qRT-PCR in this study.
