# Supplementary figures and images for: A Transcriptome Analysis of Poncirus trifoliata, an Aurantioideae Species Tolerant to Asian Citrus Psyllid, Has Identified Potential Genes and Events Associated with Psyllid Resistance
Source: Insects. 2024 Aug 2;15(8):589. doi: 10.3390/insects15080589 (PMC11354599; doi:10.3390/insects15080589)

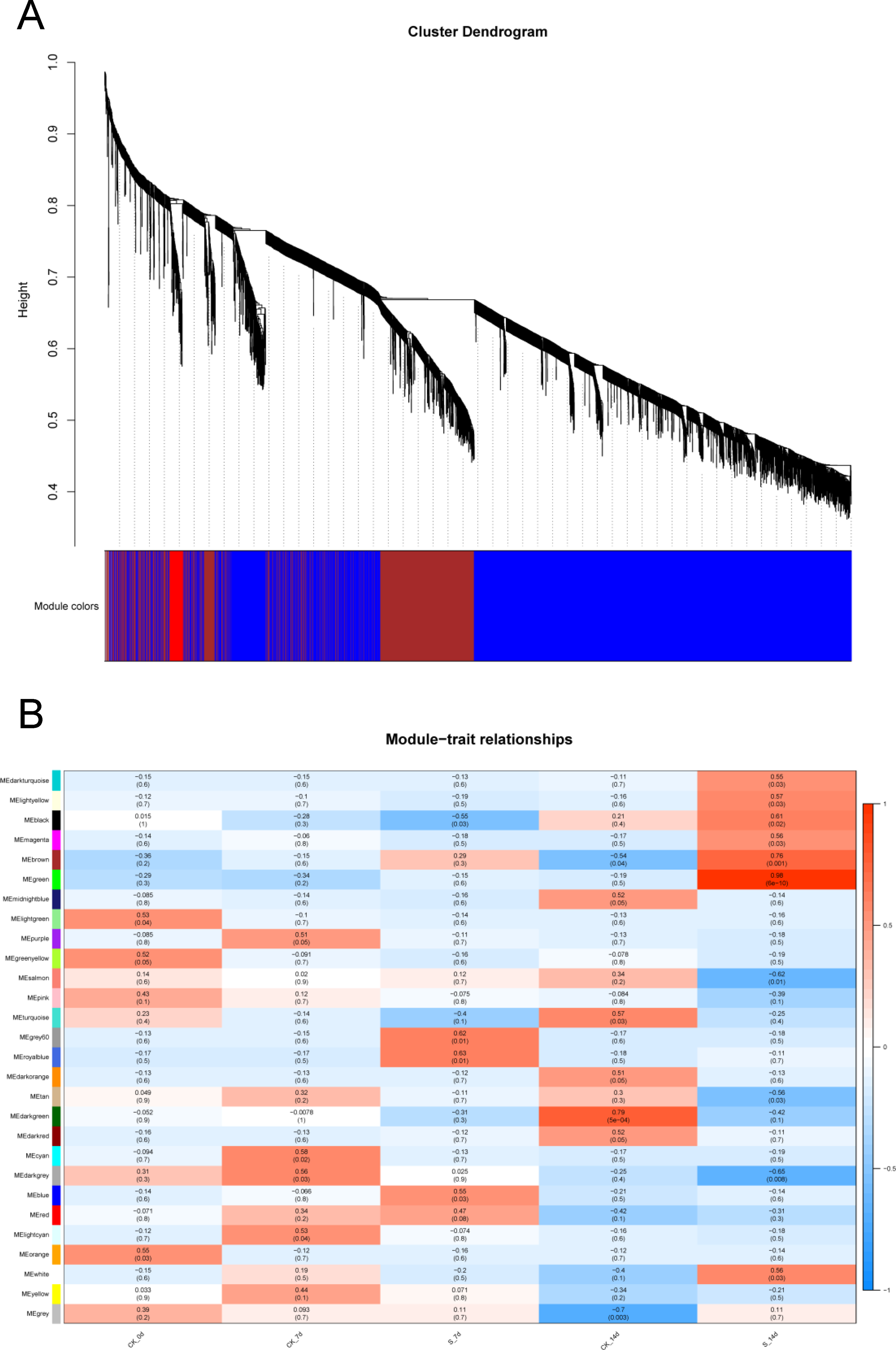

Supplement: Supplementary file 1 [file insects-15-00589-s001.zip › Figure S1-Co-expression network construction using WGCNA analysis.tif]

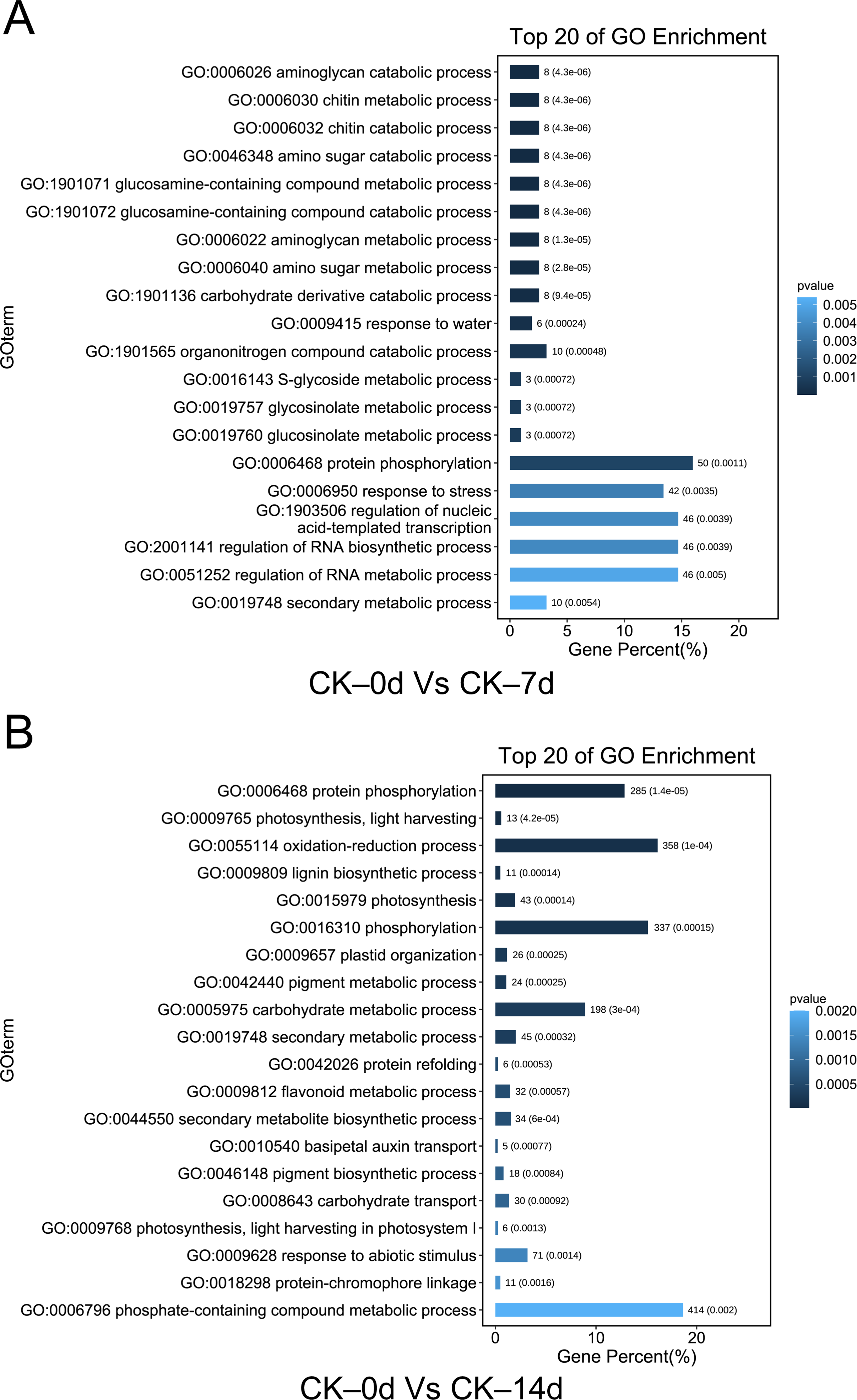

Supplement: Supplementary file 1 [file insects-15-00589-s001.zip › Figure S2-Gene ontology enrichment of differential expressed genes (DEGs) between CK samples at different points in time.tif]

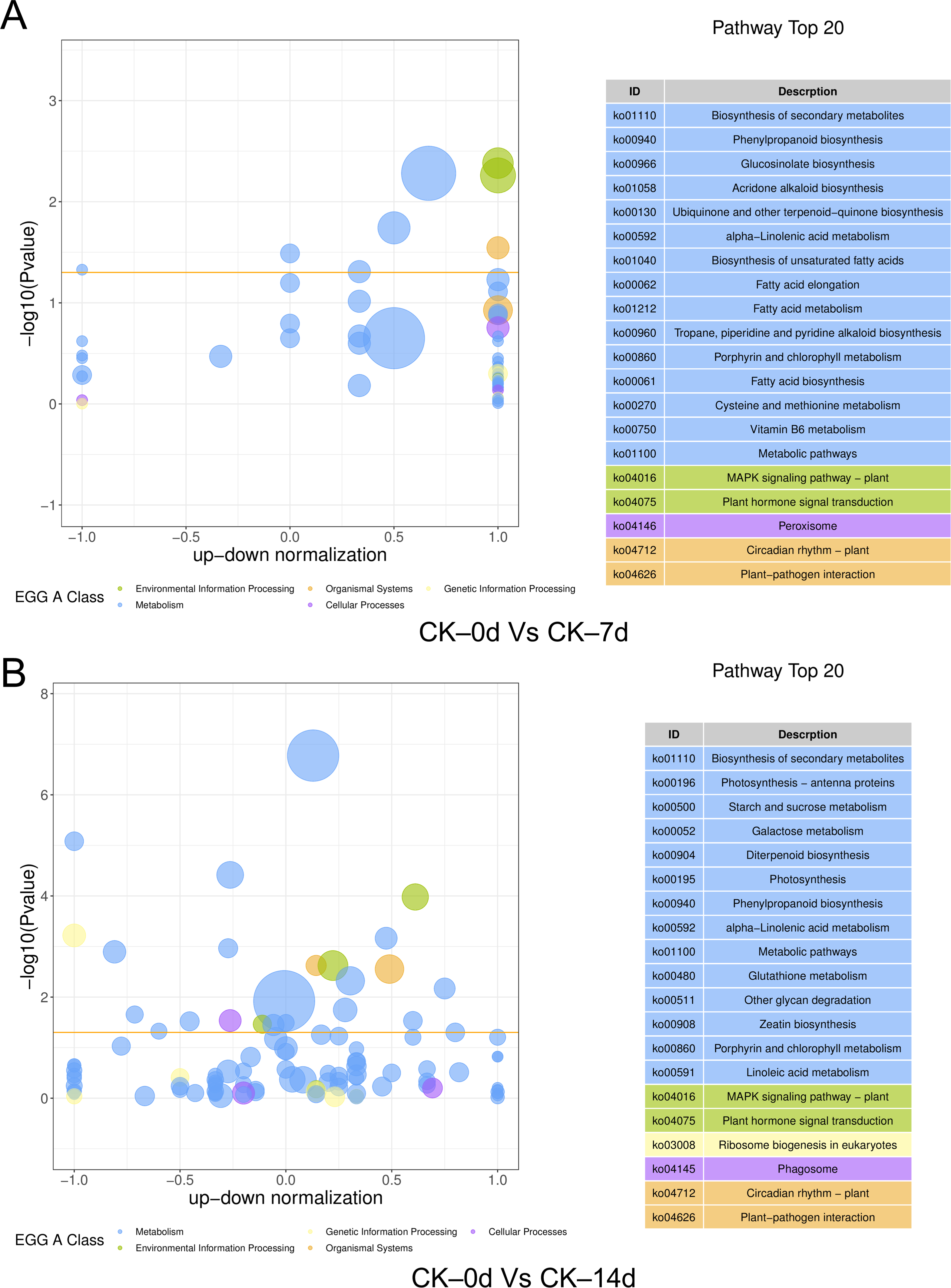

Supplement: Supplementary file 1 [file insects-15-00589-s001.zip › Figure S3-KEGG enrichment of differential expressed genes (DEGs) between CK samples at different points in time.tif]
